# A Fuzzy Computing Model for Identifying Polarity of Chinese Sentiment Words

**DOI:** 10.1155/2015/525437

**Published:** 2015-04-23

**Authors:** Bingkun Wang, Yongfeng Huang, Xian Wu, Xing Li

**Affiliations:** ^1^Department of Electronic Engineering, Tsinghua University, Beijing 100084, China; ^2^Beijing University of Posts and Telecommunications, Beijing 100876, China

## Abstract

With the spurt of online user-generated contents on web, sentiment analysis has become a very active research issue in data mining and natural language processing. As the most important indicator of sentiment, sentiment words which convey positive and negative polarity are quite instrumental for sentiment analysis. However, most of the existing methods for identifying polarity of sentiment words only consider the positive and negative polarity by the Cantor set, and no attention is paid to the fuzziness of the polarity intensity of sentiment words. In order to improve the performance, we propose a fuzzy computing model to identify the polarity of Chinese sentiment words in this paper. There are three major contributions in this paper. Firstly, we propose a method to compute polarity intensity of sentiment morphemes and sentiment words. Secondly, we construct a fuzzy sentiment classifier and propose two different methods to compute the parameter of the fuzzy classifier. Thirdly, we conduct extensive experiments on four sentiment words datasets and three review datasets, and the experimental results indicate that our model performs better than the state-of-the-art methods.

## 1. Introduction

With the advent of Web2.0, user-generated contents such as product reviews, status on social networking services, and microblogs are exploding in the Internet. The growing availability of subjective contents makes extracting useful sentiment information from subjective contents a hot topic in natural language processing, web mining, and data mining [1]. Since early 2000, as a special case of text classification, sentiment classification has attracted attention from increasing number of researchers and become a very active research area [2]. Sentiment words play a more important role in mining subjective contents than in mining objective contents [3]. As the prerequisite of sentiment classification, identifying the polarity of sentiment words is a key issue in sentiment classification.

At present, there are mainly two types of methods in identifying the polarity of English sentiment words. One is based on corpus; the other is based on thesaurus. These two methods are also widely used for identifying the polarities of Chinese sentiment words. There are mainly three steps in the two types of methods. The first step is to compute similarity between sentiment words and positive reference words. The second step is to compute similarity between sentiment words and negative reference words. The third step is to compare the two similarities based on the Cantor set and acquiring the polarity of sentiment words [4].

The existing two types of methods simply divide sentiment words into two classes, that is, positive or negative, without regarding polarity intensity of sentiment words and fuzziness of polarity intensity of sentiment words [5]. Actually, different sentiment words belonging to the same polarity have different polarity intensity. For example, “laugh” has a larger intensity than “smile.” In order to distinguish polarity intensity of different sentiment words, researchers have proposed some methods to identify polarity of Chinese sentiment words based on Chinese morphemes [6–10]. This type of methods hypothesizes that words are function of its component morphemes and can improve performance [8]. In a certain extent, this type of methods overcomes the shortcomings that the polarity intensity of sentiment words is not considered in identifying polarity of Chinese sentiment words, but the fuzziness of the polarity intensity of sentiment words is still not considered in this type of methods. Due to the fuzziness of natural language and sentiment category, we should adopt a fuzzy set to describe polarity of sentiment words instead of the Cantor set [11].

In order to overcome the above shortcomings and to improve the accuracy as best as we can, we propose a fuzzy computing model to identify the polarity of Chinese sentiment words. Our model mainly includes two parts: one is calculating polarity intensity of sentiment morphemes and sentiment words; the other is constructing a fuzzy classifier and computing parameter of the fuzzy classifier. The contribution of this paper is mainly embodied in three aspects.

Firstly, based on the three existing Chinese sentiment lexicons, we constructed an unambiguous key sentiment lexicon and a key sentiment morpheme set. Then, we proposed a method to compute the sentiment intensity of sentiment morphemes and sentiment words using the constructed sentiment lexicon and sentiment morpheme set.

Secondly, considering the fuzziness of sentiment intensity, we constructed a fuzzy sentiment classifier and a corresponding classification function of the fuzzy classifier by virtue of fuzzy sets theory and the principle of maximum membership degree. In order to improve the performance, we further proposed two different methods to learn parameters of the fuzzy sentiment classifier.

Thirdly, we constructed four sentiment words datasets to demonstrate the performance of our model. At the same time, we proved that our model performs better than several state-of-the-art methods by applying our model to sentiment classification on three review datasets.

This paper is organized as follows. We introduce related work in Section 2.  Section 3 introduces the fuzzy computing model and two key parts of the model. In Section 4, we firstly build a key sentiment lexicon, a key sentiment morpheme set, and four sentiment word datasets and then conduct some experiments to verify performance of the fuzzy computing model. Finally, we summarize this paper, draw corresponding conclusions, and figure out future research direction in Section 5.

## 2. Related Work

Sentiment classification is a hot topic in natural language processing and web mining. There are a large number of research papers about sentiment classification since 2002 [1, 2]. Existing methods are mainly divided into two categories: machine learning methods and semantic orientation aggregation [12]. The machine learning methods include many traditional text classification methods [13], such as naive Bayes [14], support vector machine [15], and neural networks [16]. The second strategy uses sentiment words to classify features into positive and negative categories and then aggregates the overall orientation of a document [17, 18].

As a basic requirement of sentiment classification, identifying the polarity of sentiment words is a research focus which has been focused on for many years. There are mainly three types of methods in identifying polarity of Chinese sentiment words. The first is thesaurus-based method which computes similarity between reference words and the given sentiment words by distance in thesaurus. The second is corpus-based method which computes similarity between reference words and the given sentiment words by statistic method in corpus. The third is morpheme-based method which computes polarity of sentiment words by combining polarity of Chinese morpheme.

Thesaurus-based method acquires sentiment words mainly by synonyms, antonyms, and hierarchies in thesaurus such as WordNet and HowNet [19–22]. These methods use some seed sentiment words to bootstrap via synonym and antonym relation in a thesaurus. Kamps et al. computed polarity of sentiment words according to the distance between sentiment words and reference seed words in WordNet [23, 24]. Esuli and Sebastiani used glosses of words to generate a feature vector and computed polarity of sentiment words with a supervised learning classifier in thesaurus [25, 26]. Dragut et al. proposed a bootstrapping method according to a set of inference rules to compute sentiment polarity of words [27].

The kernel of corpus-based method is to calculate similarity between reference words and sentiment words in corpus. This approach has an implied hypothesis that sentiment words have the same polarity with the reference words of the greatest cooccurrence rate and the opposite polarity with the reference words of the least cooccurence rate. The polarity of sentiment words is assigned by computing cooccurrence in corpus [28–35].

The most classic method is point mutual information method proposed by Turney [30, 31]. This method computes the polarity of a given sentiment word by subtracting the mutual information of its association with a set of negative sentiment words from the mutual information of its association with a set of positive sentiment words. The result of mutual information depends on statistic result in a given corpus.

Different from Turney, Yu and Hatzivassiloglou used more seed words and log-likelihood ratio to compute the similarity [32]. Kanayama and Nasukawa used a set of linguistic rules in intrasentence and intersentence to identify polarity of sentiment words from the corpus [34]. Huang et al. proposed an automatic construction of domain-specific sentiment lexicon [28]. Ghazi et al. used sentential context to identify the contextual-sensitive sentiment words [29].

Some researchers calculated polarity of sentiment words by combining corpus with thesaurus [36, 37]. Xu et al. presented a method to capture polarity of sentiment words by using a graph-based algorithm and multiple resources [36]. Peng and Park computed polarity of sentiment words by constrained symmetric nonnegative matrix factorization [37]. This method finds out a set of candidate sentiment words by bootstrapping in dictionary and then uses a large corpus to assign sentiment polarity scores to each word.

Taking into consideration the characteristics of Chinese character, some researchers proposed morpheme-based methods [6–10]. Based on Turney's work, Yuen et al. proposed a method by calculating similarity between reference morphemes and sentiment words in corpus to get the polarity of sentiment words [6]. Experimental results demonstrated better performance than Turney's method in identifying polarity of Chinese sentiment words. Ku et al. proposed a bag-of-characters method, which computed polarity intensity of sentiment words based on morpheme by statistic and then compared polarity intensity of sentiment words with a single threshold “0” to identify polarity of sentiment words [10]. Ku et al. considered eight types of morphemes and built a classifier based on machine learning for Chinese word-level sentiment classification [8]. They showed that using word structural features can improve performance in word-level sentiment classification.

The existing three types of methods are based on a common hypothesis that the polarity of sentiment words is certainty. But some researches have proven that polarity of sentiment words had some fuzziness to some extent [5]. So, it is not suitable to identify polarity of sentiment words by either-or methods. To this end, we propose a fuzzy computing model to identify polarity of Chinese sentiment words.

Some researches on fuzzy set have been applied to sentiment classification. These researches mainly focus on document-level and sentence-level sentiment classification [9, 12]. For example, Wang et al. proposed an ensemble learning method to predict consumer sentiment by online sequential extreme learning machine and intuitionist fuzzy set, which is a supervised method [12]. Fu and Wang together invented an unsupervised method using fuzzy sets for sentiment classification of Chinese sentences [9]. Different from the above methods, we focus on word-level sentiment classification and propose a fuzzy computing model, which is an unsupervised framework to identify the polarity of Chinese sentiment words.

## 3. Fuzzy Computing Model

### 3.1. General Framework

**Figure 1:**
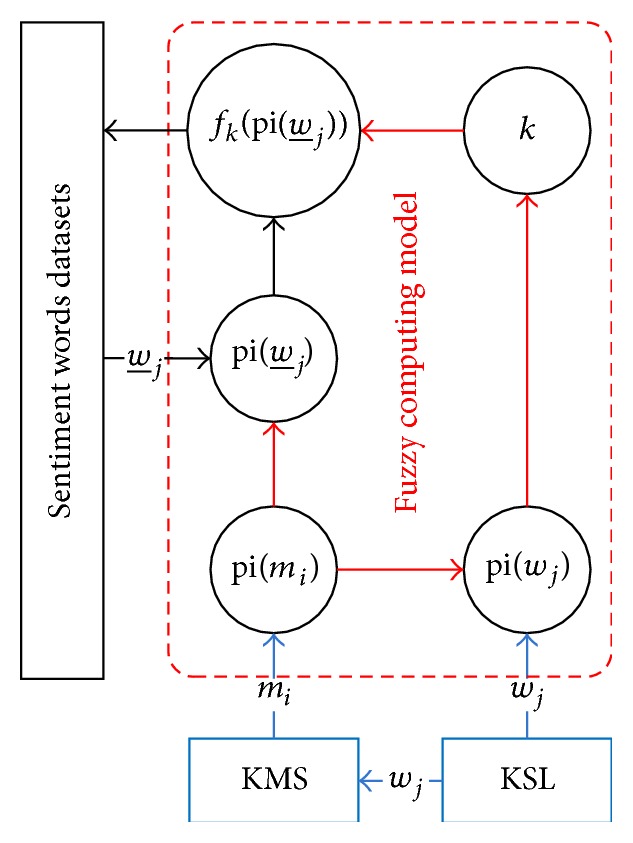
General framework of fuzzy computing model.

In existing methods of identifying the polarity of sentiment words, sentiment words are divided into two classes—positive or negative by Cantor set. The fuzziness of polarity intensity of sentiment words is not considered. In order to overcome the shortcomings and improve the accuracy, we proposed a fuzzy computing model (FCM) for identifying polarity of Chinese sentiment words. The general framework of FCM is described in Figure 1.

Notations KSL, KMS, *m*_*i*_, *w*_*j*_, pi(*m*_*i*_), pi(*w*_*j*_), ${\underline{w}}_{j}$, $\text{pi}({\underline{w}}_{j})$, *k*, and $f_{k}(\text{pi}({\underline{w}}_{j}))$ in Figure 1 are defined in Notations section.

The general framework of FCM consists of three sections: sentiment words datasets, a key sentiment lexicon (KSL) and a key sentiment morpheme set (KMS), and the central FCM. Sentiment words datasets are test datasets for verifying the performance of FCM when identifying the polarity of Chinese sentiment words. KSL and KMS are the basic thesauruses of FCM. KSL consists of a positive key sentiment words list (P_KSL) and a negative key sentiment words list (N_KSL). The central FCM is composed of two key parts.

The first part includes computing polarity intensity pi(*m*_*i*_) of sentiment morpheme *m*_*i*_ in KMS, computing polarity intensity pi(*w*_*j*_) of sentiment word *w*_*j*_ in KSL, and computing polarity intensity $\text{pi}({\underline{w}}_{j})$ of sentiment word ${\underline{w}}_{j}$ in sentiment words datasets. We compute pi(*m*_*i*_) based on the frequency of sentiment morpheme *m*_*i*_ appearing in P_KSL and the frequency of sentiment morpheme *m*_*i*_ appearing in N_KSL. After getting pi(*m*_*i*_), we divide each sentiment word into morphemes and compute pi(*w*_*j*_) and $\text{pi}({\underline{w}}_{j})$ based on pi(*m*_*i*_).

The second part is to construct a classification function $f_{k}(\text{pi}({\underline{w}}_{j}))$ of fuzzy classifier and computing parameter *k* in $f_{k}(\text{pi}({\underline{w}}_{j}))$. Firstly, we define fuzzy set and membership function of fuzzy set for positive or negative categories. Secondly, based on the principle of maximum membership degree, we construct $f_{k}(\text{pi}({\underline{w}}_{j}))$. Thirdly, we propose two different methods based on average polarity intensity of sentiment words (APIOSW) in different sentiment word datasets and APIOSW in KSL to determine *k*. Then, we describe the two key components of FCM in detail.

### 3.2. Computing Polarity Intensity of Sentiment Morphemes and Sentiment Words

Based on KSL and KMS, we calculate pi(*m*_*i*_) in KMS. With pi(*m*_*i*_) available, we compute pi(*w*_*j*_) in KSL and $\text{pi}({\underline{w}}_{j})$ in sentiment words datasets. There are mainly three steps in the whole computational process.

(1) Firstly, for each sentiment morpheme *m*_*i*_ in KMS, we compute positive frequency of *m*_*i*_ appearing in P_KSL and negative frequency of *m*_*i*_ appearing in N_KSL according to
  (1) fmi ∣ P_KSLnumbermi,P_KSLnumberP_KSL,fmi ∣ N_KSL=numbermi,N_KSLnumberN_KSL.
 Here *f*(*m*_*i*_∣P_KSL) is the frequency of *m*_*i*_ appearing in P_KSL and *f*(*m*_*i*_∣N_KSL) is the frequency of *m*_*i*_ appearing in N_KSL. number(*m*_*i*_, P_KSL) is the number of positive sentiment words that contain the morpheme *m*_*i*_ and number(*m*_*i*_, N_KSL) is the number of negative sentiment words that contain the morpheme *m*_*i*_. number(P_KSL) is the number of sentiment words in P_KSL and number(N_KSL) is the number of sentiment words in N_KSL.
(2) Secondly, for each sentiment morpheme *m*_*i*_ in KMS, we use percentage of *m*_*i*_ in *f*(*m*_*i*_∣P_KSL) and *f*(*m*_*i*_∣N_KSL) to compute positive polarity intensity, negative polarity intensity, and polarity intensity by
  (2) pmifmi ∣ P_KSLfmi ∣ P_KSL+fmi ∣ N_KSLnmi=fmi ∣ N_KSLfmi ∣ P_KSL+fmi ∣ N_KSLpimi=pmi−nmi.
 Here *p*(*m*_*i*_) is positive polarity intensity, *n*(*m*_*i*_) is negative polarity intensity, and pi(*m*_*i*_) is polarity intensity. *f*(*m*_*i*_∣P_KSL) is the frequency of *m*_*i*_ appearing in P_KSL and *f*(*m*_*i*_∣N_KSL) is the frequency of *m*_*i*_ appearing in N_KSL.
(3) Thirdly, for each sentiment word *w*_*j*_ in KSL and ${\underline{w}}_{j}$ in sentiment words datasets, we calculate pi(*w*_*j*_) and $\text{pi}({\underline{w}}_{j})$ based on polarity intensity of *m*_*i*_ by
  (3) piwj1numbermi,wj∑i=1numbermi,wjpimi,piw_j=1numbermi,w_j∑i=1numbermi,w_jpimi.
 Here number(*m*_*i*_, *w*_*j*_) is the number of morphemes *m*_*i*_ included in sentiment words *w*_*j*_; number(*m*_*i*_, ${\underline{w}}_{j}$) is the number of morphemes *m*_*i*_ included in sentiment words ${\underline{w}}_{j}$.

### 3.3. Constructing Fuzzy Classifier and Computing Parameter of Fuzzy Classifier

After getting pi(*m*_*i*_), pi(*w*_*j*_), and $\text{pi}({\underline{w}}_{j})$, we firstly define two membership functions of the fuzzy classifier for positive and negative categories. Secondly, we build a classification function of the fuzzy classifier by principle of maximum membership degree. Thirdly, we determine an optimum fixed parameter of the fuzzy classifier by experimenting on KSL. Fourthly, we compare APIOSW in different sentiment word datasets with APIOSW in KSL to get different optimum parameters of fuzzy classifier for each sentiment word dataset.

#### 3.3.1. Defining Membership Function of Fuzzy Classifier

In order to identify polarity of sentiment words based on FCM, we choose semitrapezoid distribution to define membership function of positive category and negative category in fuzzy classifier by

(4) μPw0,piw<αx−αβ−α,α<piw<β1,piw>β,μNw=1,piw<αβ−xβ−α,α<piw<β0,piw>β.

Here *w* is sentiment word, pi(*w*) is polarity intensity of *w*, and *α*, *β* are adjustable parameters which decide the region and the shape of member function of the fuzzy classifier.

We need to set value for parameters *α*, *β* in membership function of the fuzzy classifier to identify polarity of sentiment words. Actually, we do not set value for the two parameters *α*, *β* in FCM but simplify the two parameters to one parameter *k* by defining *k* = (*α* + *β*)/2.

#### 3.3.2. Constructing Classification Function of Fuzzy Classifier

After computing polarity intensity of sentiment words, based on membership function in (4), we confirm polarity of sentiment words according to the principle of maximum membership degree. At last, we get the following equation as the classification function of the fuzzy classifier to identify polarity of sentiment words:

(5) fkpiwmax⁡μPw,μNw=w∈N,piw<kw∈P,piw>k.

Here pi(*w*) is polarity intensity of sentiment word *w*. We can determine polarity of sentiment words only by setting value for parameter *k*.

#### 3.3.3. Setting Parameter *k* in Classification Function of the Fuzzy Classifier

In order to determine the parameter *k* in classification function of the fuzzy classifier, we propose two different methods. One is fixed parameter method where the parameter is selected by experimenting on KSL; the other is variable parameter method where the parameter is selected by comparing APIOSW of different sentiment word datasets with APIOSW of KSL. The two methods are described as follows.

**Figure 2:**
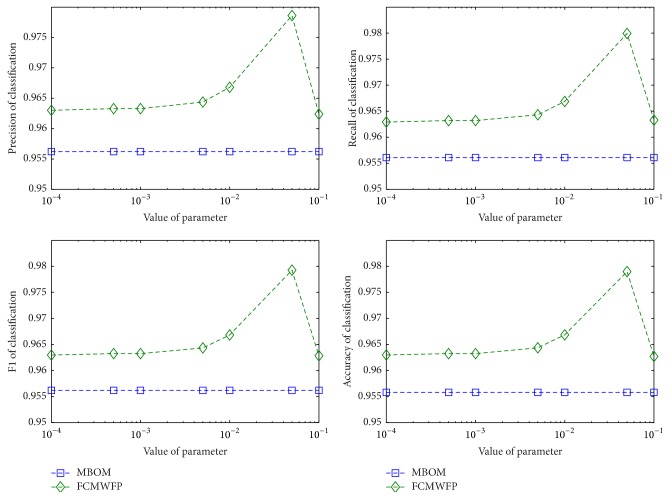
Performance of MBOM and FCMWFP in different parameter *k* in KSL.

*(1) Fixed Parameter Method*. The parameter *k* controls the threshold of identifying polarity of sentiment words in fuzzy classifier. We can choose parameter *k* by experimenting on KSL. We compare performance of FCM with different values of parameter *k* and then choose the best-performance parameter *k*_0_ as fixed parameter in FCM. The experimental results and specific parameter *k*_0_ are shown in Figure 2. 

*(2) Variable Parameter Method*. Referring to experiment results in KSL to estimate the value of parameter *k*, we can only get a local optimal parameter *k*_0_ in KSL. In order to get global optimal parameter *k* in different sentiment word datasets, we propose a variable parameter method. The specific method is described as follows.

For each sentiment word datasets (SWD_*i*_) which consist of positive sentiment words list P_SWD_*i*_ and negative sentiment words list N_SWD_*i*_, we define APIOSW_*i*_ in

(6) $$\begin{array}{c} \text{APIOS}{\text{W}}_{i}=\frac{1}{M}\overset{M_{i}}{\underset{j=1}{\sum }}\text{pi}\left(\;{\underline{w}}_{ij}\;\right)+\frac{1}{N-M}\overset{N_{i}-M_{i}}{\underset{j=1}{\sum }}\text{pi}\left(\;{\underline{w}}_{ij}\;\right). \end{array}$$

Here *M*_*i*_ is the number of sentiment words in *P*_SWD_*i*_, *N*_*i*_ is the number of sentiment words in SWD_*i*_, and $\text{pi}({\underline{w}}_{ij})$ is polarity intensity of sentiment word ${\underline{w}}_{ij}$ in SWD_*i*_. Similar to SWD_*i*_, we define APIOSW of KSL in

(7) $$\begin{array}{c} \text{APIOS}{\text{W}}_{\text{KSL}}=\frac{1}{M}\overset{M}{\underset{j=1}{\sum }}\text{pi}\left(\;w_{j}\;\right)+\frac{1}{N-M}\overset{N-M}{\underset{j=1}{\sum }}\text{pi}\left(\;w_{j}\;\right). \end{array}$$

Here *M* is the number of sentiment words in P_KSL, *N* is the number of sentiment words in KSL, and pi(*w*_*j*_) is polarity intensity of sentiment word *w*_*j*_ in KSL.

For each SWD_*i*_, we calculate the difference of APIOSW between SWD_*i*_ and KSL. Based on the difference and fixed parameter *k*_0_ in KSL, we adjust the parameter of SWD_*i*_ to get different optimum parameter *k*_*i*_ in each SWD_*i*_. The special method is described in

(8) $$\begin{array}{c} k_{i}=\frac{\text{APIOS}{\text{W}}_{i}-\text{APIOS}{\text{W}}_{\text{KSL}}}{\text{APIOS}{\text{W}}_{\text{KSL}}}\times k_{0}. \end{array}$$

Here parameter *k*_0_ is a fixed parameter which is got through the fixed parameter method above.

## 4. Performance Evaluation

To verify the performance of FCM by experiment, we firstly construct KSL and KMS. Secondly, we construct four sentiment word datasets as test datasets and choose classification indicator: precision, recall, *F*1 measure, and accuracy as metric to evaluate the performance of baseline methods and FCM. Thirdly, we do experiments on KSL and compare APIOSW in different sentiment word datasets with APIOSW in KSL to find the optimum parameter. Fourthly, we compare the performance of different methods and prove the efficiency of FCM. Fifthly, we discuss the influence of parameter *k* on accuracy of FCM. Sixthly, in order to demonstrate the effect of our methods in a real task, we apply our methods and baseline methods to sentiment classification of review. The experimental results prove the validity of our method.

### 4.1. Constructing KSL and KMS

Based on Chinese sentiment lexicons—Tsinghua University sentiment lexicon (TUSL), National Taiwan University sentiment lexicon (NTUSL), and HowNet, we construct KSL. When constructing KSL, we have an implicit assumption that there are some sentiment words whose polarity is ambiguous among different sentiment lexicons. We define TUSL as SL_1_, NTUSL as SL_2_, and HowNet as SL_3_. Given above SL_*i*_ which consists of P_SL_*i*_ and N_SL_*i*_, we get some sentiment words whose polarity is ambiguous.  Table 1 presents the number of sentiment words whose polarity is fuzzy between P_SL_*i*_ and N_SL_*i*_.

From Table 1, we can see that there are some sentiment words whose polarity is ambiguous between P_SL_*i*_ and N_SL_*i*_, which proves the assumption that polarity of sentiment words is not always consistent within different sentiment lexicons. So, we delete these sentiment words whose polarity is ambiguous from P_SL_*i*_ and N_SL_*i*_. Finally, we construct KSL by choosing the sentiment words which is at least contained in two sentiment lexicons and unambiguous in polarity. The method of constructing KSL is shown in

(9) P_KSL=⋃i<jP_SLi∩P_SLj−⋃i<jP_SLi∩P_SLj∩⋃i<jN_SLi∩N_SLj,N_KSL=⋃i<jN_SLi∩N_SLj−⋃i<jP_SLi∩P_SLj∩⋃i<jN_SLi∩N_SLj.

Here *i* = 1,2, 3, *j* = 1,2, 3, P_SL_*i*_ is positive sentiment words list of SL_*i*_, and N_SL_*i*_ is negative sentiment words list of SL_*i*_. We compute KSL in (9). The number of sentiment words in SL_*i*_ and KSL is shown in Table 2.

For KSL, we delete words whose length is greater than two and then split the remaining words into morphemes. Finally, we put morphemes together to construct a KMS.

### 4.2. Experimental Setting

In order to verify the performance of our model, we build four sentiment word datasets based on TUSL, NTUSL, and HowNet. To ensure that the polarity of sentiment words is unambiguous in the four sentiment word datasets, we delete the sentiment words whose polarity is ambiguous among the three sentiment lexicons. In order to ensure that sentiment words in the four sentiment word datasets is independent of sentiment words in KSL, we delete the sentiment words in KSL from the three sentiment lexicons. The specific method is described as follows.

For each SL_*i*_ which consists of P_SL_*i*_ and N_SL_*i*_, we build sentiment words dataset1, dataset2 and dataset3 according to

(10) P_DSiP_SLi−P_SLi∩N_SLi−P_KSL,N_DSi=N_SLi−P_SLi∩N_SLi−N_KSL.

With the same method, we construct a much larger datasets4 in

(11) P_DS4=−P_KSL+⋃i=13P_SLi−⋃i=13P_SLi∩⋃i=13N_SLi,N_DS4=−N_KSL+⋃i=13N_SLi−⋃i=13P_SLi∩⋃i=13N_SLi.

Each sentiment word dataset SWD_*i*_ consists of positive sentiment words list P_SWD_*i*_ and negative sentiment words list N_SWD_*i*_. At last, we get four sentiment word datasets (http://203.91.121.76/Datasets/) which are summarized in Table 3.

Since our task is identifying polarity of sentiment words, therefore, we choose classification indicator: precision (*P*), recall (*R*), *F*1 measure, and accuracy (AC⁡) as metric. The four indicators are defined as follows:

(12) Macro_P12R1R1+W2+R2R2+W1,Macro_R=12R1R1+W1+R2R2+W2,Macro_F1=2×Macro_P×Macro_RMacro_P+Macro_R,Acuracy=R1+R2W1+W2+R1+R2.

Here *R*_1_, *R*_2_, *W*_1_, and *W*_2_ are defined in Table 4.

To evaluate the overall performance of our model in identifying the polarity of Chinese sentiment words, we compare our model with thesaurus-based method and morpheme-based method in four different sentiment word datasets. Our model and baseline methods are depicted as follows:

 MBOT: the method based on thesaurus [27];
 MBOM: the method based on morpheme [10];
 FCMWFP: the fuzzy computing model with fixed parameter, which is described in Section 3.3.3;
 FCMWVP: the fuzzy computing model with variable parameter, which is shown in Section 3.3.3.

In order to further demonstrate the effect of our methods and highlight the contribution of our work, we design the following experiments. Firstly, for each sentiment word dataset, we construct four different sentiment lexicons where the polarities of sentiment words are different in each method. Secondly, we choose three Chinese review datasets, which are provided by Songbo Tan (http://203.91.121.76/Datasets/). Each review dataset (RDS_*i*_) consists of both positive reviews and negative reviews. The basic statistics of these three review datasets are summarized in Table 5. Thirdly, for each sentiment word dataset, we compare sentiment classification results of three review datasets based on four different sentiment lexicons, which correspond to four different methods. These sentiment lexicons are described as follows:

 SLMBOT_*i*_: the sentiment lexicon corresponding to MBOT and sentiment word dataset *i*;
 SLMBOM_*i*_: the sentiment lexicon corresponding to MBOM and sentiment word dataset *i*;
 SLFCMWFP_*i*_: the sentiment lexicon corresponding to FCMWFP and sentiment word dataset *i*;
 SLFCMWVP_*i*_: the sentiment lexicon corresponding to FCMWVP and sentiment word dataset *i*.

We conduct extensive experiments in four sentiment word datasets and three review datasets to solve four problems.

1. Discuss how to set parameter *k* in classification function of fuzzy classifier.
2. Study performance of our model in identifying polarity of Chinese sentiment words.
3. Analyse effect of different parameter *k* on accuracy of our model.
4. Validate the effect of sentiment lexicons created by our methods in sentiment classification of documents.

### 4.3. Setting Parameter *k* in Classification Function of Fuzzy Classifier

In FCM, parameter *k* in classification function of fuzzy classifier needs to be set. We conducted experiment on KSL to find the optimal value of parameter *k*. Figure 2 shows performance of MBOM and FCMWFP for different parameter *k*.

From Figure 2, we can see that when parameter *k* is selected near 0.05, performance of FCMWFP is the best. So we choose *k* = 0.005 in FCMWFP. After choosing the fixed value *k*_0_ of parameter *k*, according to Section 3.3.3, we calculate APIOSW of different datasets by (6) and (7). Finally, we compute value of parameter *k*_*i*_ by (8).  Table 6 summarizes the APIOSW and *k*_*i*_ of KSL and four sentiment word datasets.

### 4.4. Performance of Different Methods in Identifying Polarity of Chinese Sentiment Words

**Figure 3:**
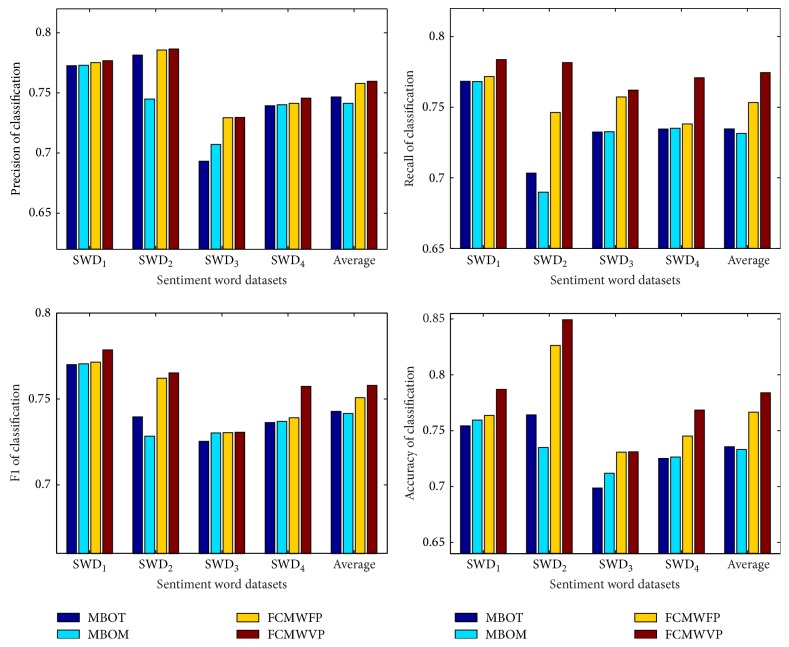
Performances of MBOT, MBOM, and FCM.

In order to verify performance of FCM, we choose MBOT and MBOM as baselines. FCM consists of FCMWFP and FCMWVP. We compared FCM with MBOT and MBOM in four sentiment word datasets. Experimental results are shown in Table 7 and Figure 3.

From Table 7 and Figure 3, we can see that accuracy of MBOT is slightly higher than accuracy of MBOM. The average accuracies of MBOT and MBOM are 0.7351 and 0.7327 in four datasets, while the average accuracies of our model are 0.7661 and 0.7835. FCMWFP improves by about 4.6% ((0.7661 − 0.7327)/0.7327) accuracy than MBOM in identifying polarity of Chinese sentiment words. FCMWVP improves by about 6.9% ((0.7835 − 0.7327)/0.7327) accuracy than MBOM in identifying polarity of Chinese sentiment words. This demonstrates that our model is more effective than MBOM and MBOT in identifying polarity of Chinese sentiment words.

At the same time, we can see that FCMWVP has better performance than FCMWFP in our model, which validates our assumption that we can only acquire local optimum by FCMWFP, but we can get approximate global optimum by FCMWVP.

### 4.5. Accuracy of FCM with Different Parameter *k*

**Figure 4:**
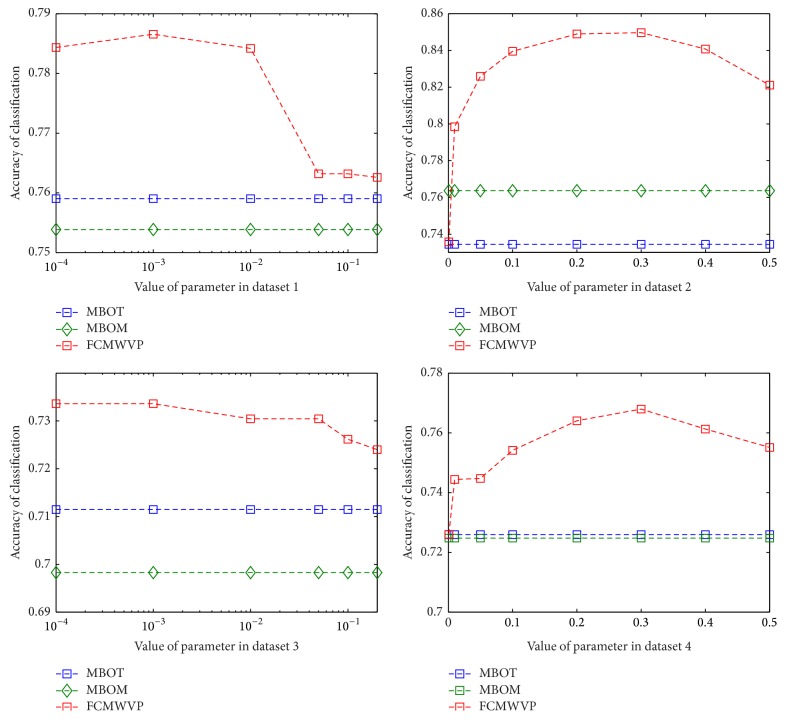
Accuracy of FCM in different parameter *k*.

In order to explore the effect of the different parameter *k* on accuracy of FCM in identifying polarity of Chinese sentiment words, we do some experiments using different parameter *k* in four sentiment word datasets. The results are shown in Figure 4.

From Figure 4, we can see that different values of parameter *k* in FCM have different effect on the accuracy of FCM. When we choose suitable parameter *k*, FCM always achieves higher accuracy than MBOT and MBOM in identifying polarity of Chinese sentiment words.

### 4.6. Performance of Different Sentiment Lexicons in Sentiment Classification of Chinese Reviews

In order to further verify the feasibility of our methods, we applied four different sentiment lexicons to sentiment classification of Chinese reviews. For each sentiment word dataset, we compared sentiment classification results of three review datasets based on four different sentiment lexicons. Experimental results are shown in Tables 8, 9, 10, and 11.

From Tables 8, 9, 10, and 11, we can see that accuracies of SLFCMWVP_*i*_ and SLFCMWFP_*i*_ are higher than accuracies of SLMBOM_*i*_ and SLMBOT_*i*_ in sentiment classification of Chinese review. The results prove that the methods which consider fuzzy sentiment are more effective than those methods that consider only either-or sentiment.

At the same time, we can see that SLFCMWVP_*i*_ had better performance than SLFCMWFP_*i*_ in sentiment classification of Chinese reviews, which proves that our method based on variable parameters was more efficient than our method based on fixed parameter.

## 5. Conclusion

In this paper, we propose a fuzzy computing model for identifying polarity of Chinese sentiment words by combining polarity intensity of Chinese morpheme with fuzzy set theory. Based on the assumption that Chinese sentiment words are a function of Chinese morpheme, we compute polarity intensity of sentiment words with known polarity intensity of morphemes. After studying the three existing sentiment lexicon, we find that there is fuzziness among some of the sentiment words; that is to say, some sentiment words have different sentiment polarities in different lexicons. We define polarity of sentiment words as fuzzy set and identify polarity of sentiment words by the principle of maximum membership degree. In order to verify performance of our model, we build four sentiment word datasets. We compare our model with baseline methods in four sentiment word datasets. Experimental results prove that our model had better performance than the state-of-the-art methods.

Our methods suggest several possible research directions. Due to fuzziness of sentiment polarity in natural language, we can deal with sentiment analysis problem based on fuzzy set theory. Our model demonstrates the effectiveness of fuzzy computing in sentiment words classification. Next, we plan to apply fuzzy set theory to sentence-level sentiment classification and document-level sentiment classification.

## Figures and Tables

**Table 1:** Number of fuzzy sentiment words between P_SL_*i*_ and N_SL_*i*_.

Reviews datasets	N_SL_1_	N_SL_2_	N_SL_3_
P_SL_1_	1	48	47
P_SL_2_	19	49	27
P_SL_3_	70	87	102

**Table 2:** Number of sentiment words in SL_*i*_ and KSL.

Sentiment lexicons	SL_1_	SL_2_	SL_3_	KSL
Number of positive sentiment words	5567	2810	4566	2015
Number of negative sentiment words	4468	8276	4370	1942

**Table 3:** Distribution of four sentiment words datasets.

SWD	SWD_1_	SWD_2_	SWD_3_	SWD_4_
Number of positive sentiment words	3918	1749	2988	8326
Number of negative sentiment words	2834	6529	3121	11771

**Table 4:** Definition of four parameters *R*_1_, *R*_2_, *W*_1_, and *W*_2_.

	Positive sentiment in datasets	Negative sentiment in datasets
Identifying positive sentiment	*R*_1_ = true positive	*W*_1_ = false positive
Identifying negative sentiment	*W*_2_ = false negative	*R*_2_ = true negative

**Table 5:** Distribution of three review datasets.

Review datasets	RDS_1_	RDS_2_	RDS_3_
Number of positive reviews	2000	2000	2000
Number of negative reviews	2000	2000	2000

**Table 6:** APIOSW and parameter *k*_*i*_ of KSL and four sentiment word datasets.

Method	KSL	SWD_1_	SWD_2_	SWD_3_	SWD_4_
APIOSW	−0.065	−0.0676	−0.299	−0.0676	−0.403
Parameter *k*_*i*_	0.05	0.002	0.18	0.002	0.26

**Table 7:** Classification result of MBOT, MBOM, and FCM in terms of confusion matrices.

		P_SWD_1_	N_SWD_1_	P_SWD_2_	N_SWD_2_	P_SWD_3_	N_SWD_3_	P_SWD_4_	N_SWD_4_
MBOT	I_P	2569	313	1404	1586	1310	169	6865	4070
MBOT	I_N	1349	2521	324	4768	1659	2920	1461	7701

MBOM	I_P	2687	396	1452	1870	1458	237	6855	4037
MBOM	I_N	1231	2438	276	4484	1511	2852	1471	7734

FCMWFP	I_P	2764	445	1240	919	2016	680	6010	2814
FCMWFP	I_N	1154	2389	488	5435	953	2409	2316	8957

FCMWVP	I_P	3324	847	973	466	2017	680	5128	1465
FCMWVP	I_N	594	1987	755	5888	952	2409	3198	10306

I_P means identifying positive sentiment; I_N means identifying negative sentiment.

**Table 8:** Performance of four different sentiment lexicons for sentiment word datasets1.

		RDS_1_	RDS_2_	RDS_3_	Average
SLMBOT_1_	*P*	0.6240	0.6575	0.57125	0.6176
	*R*	0.6469	0.6659	0.5808	0.6312
	*F*1	0.6352	0.6617	0.5760	0.6243
	AC	0.6240	0.6575	0.57125	0.6176

SLMBOM_1_	*P*	0.6270	0.65975	0.5760	0.6209
	*R*	0.6550	0.6666	0.5843	0.6353
	*F*1	0.6407	0.6632	0.5801	0.628
	AC	0.6270	0.65975	0.5760	0.6209

SL-FCMWFP_1_	*P*	0.63175	0.7105	0.60525	0.6492
	*R*	0.6409	0.7180	0.6160	0.6583
	*F*1	0.6363	0.7143	0.6106	0.6537
	AC	0.63175	0.7105	0.60525	0.6492

SL-FCMWVP_1_	*P*	0.63225	0.71075	0.6060	0.6497
	*R*	0.6556	0.7183	0.6167	0.6635
	*F*1	0.6437	0.7145	0.6113	0.6565
	AC	0.63225	0.71075	0.6060	0.6497

**Table 9:** Performance of four different sentiment lexicons for sentiment word datasets2.

		RDS_1_	RDS_2_	RDS_3_	Average
SLMBOT_2_	*P*	0.62375	0.64675	0.6365	0.6357
	*R*	0.6542	0.6906	0.6936	0.6795
	*F*1	0.6386	0.6680	0.6638	0.6568
	AC	0.62375	0.64675	0.6365	0.6357

SLMBOM_2_	*P*	0.6270	0.66325	0.65075	0.6470
	*R*	0.6552	0.6985	0.7017	0.6851
	*F*1	0.6408	0.6804	0.6753	0.6655
	AC	0.6270	0.66325	0.65075	0.6470

SL-FCMWVP_2_	*P*	0.68575	0.67375	0.6645	0.6747
	*R*	0.6968	0.6961	0.7042	0.6990
	*F*1	0.6912	0.6847	0.6838	0.6866
	AC	0.68575	0.67375	0.6645	0.6747

SL-FCMWVP_2_	*P*	0.7045	0.6800	0.66975	0.6848
	*R*	0.7183	0.6940	0.7058	0.7060
	*F*1	0.7113	0.6869	0.6873	0.6952
	AC	0.7045	0.6800	0.66975	0.6848

**Table 10:** Performance of four different sentiment lexicons for sentiment word datasets3.

		RDS_1_	RDS_2_	RDS_3_	Average
SLMBOT_3_	*P*	0.6113	0.6780	0.6110	0.6334
	*R*	0.6152	0.6782	0.6178	0.6371
	*F*1	0.6132	0.6781	0.6144	0.6352
	AC	0.6113	0.6780	0.6110	0.6334

SLMBOM_3_	*P*	0.6205	0.6808	0.6133	0.6382
	*R*	0.6260	0.6809	0.6206	0.6425
	*F*1	0.6232	0.68085	0.6169	0.6403
	AC	0.6205	0.6808	0.6133	0.6382

SL-FCMWFP_3_	*P*	0.6463	0.7333	0.6575	0.6790
	*R*	0.6513	0.7335	0.6672	0.6840
	*F*1	0.6488	0.7334	0.6623	0.6815
	AC	0.6463	0.7333	0.6575	0.6790

SL-FCMWVP_3_	*P*	0.6470	0.7350	0.6603	0.6808
	*R*	0.6526	0.7352	0.6688	0.6855
	*F*1	0.6498	0.7351	0.6645	0.6831
	AC	0.6470	0.7350	0.6603	0.6808

**Table 11:** Performance of four different sentiment lexicons for sentiment word datasets4.

		RDS_1_	RDS_2_	RDS_3_	Average
SLMBOT_4_	*P*	0.4855	0.6315	0.5648	0.5606
	*R*	0.4853	0.6365	0.5720	0.5646
	*F*1	0.4854	0.6340	0.5684	0.5626
	AC	0.4855	0.6315	0.5648	0.5606

SLMBOM_4_	*P*	0.5010	0.6395	0.58275	0.5744
	*R*	0.5010	0.6421	0.5841	0.5757
	*F*1	0.5010	0.6408	0.5835	0.5751
	AC	0.5010	0.6395	0.58275	0.5744

SL-FCMWFP_4_	*P*	0.5445	0.6735	0.6058	0.6079
	*R*	0.5449	0.6758	0.6125	0.6111
	*F*1	0.5447	0.6746	0.6091	0.6095
	AC	0.5445	0.6735	0.6058	0.6079

SL-FCMWVP_4_	*P*	0.5453	0.6785	0.6060	0.6099
	*R*	0.5456	0.6789	0.6001	0.6082
	*F*1	0.5454	0.6787	0.6030	0.6090
	AC	0.5453	0.6785	0.6060	0.6099

## Notations

KMS: Key sentiment morpheme
KSL: Key sentiment lexicon
*m*_*i*_: Sentiment morpheme in KMS
*w*_*j*_: Sentiment word in KSL
pi(*m*_*i*_): Polarity intensity of sentiment morpheme *m*_*i*_ in KMS
pi(*w*_*j*_): Polarity intensity of sentiment word *w*_*j*_ in KSL
${\underline{w}}_{j}$: Sentiment word in sentiment words datasets
$\text{pi}({\underline{w}}_{j})$: Polarity intensity of sentiment word ${\underline{w}}_{j}$ in sentiment words datasets
*k*: Parameter in classification function of fuzzy classifier
$f_{k}(\text{pi}({\underline{w}}_{j}))$: Classification function of fuzzy classifier.
